# Comparative immunogenicity assessment of biosimilar natalizumab to its reference medicine: a matching immunogenicity profile

**DOI:** 10.3389/fimmu.2024.1414304

**Published:** 2024-12-19

**Authors:** Paul Chamberlain, Bernhard Hemmer, Josef Höfler, Hendrik Wessels, Oliver von Richter, Cyrill Hornuss, Johann Poetzl, Karsten Roth

**Affiliations:** ^1^ bioLOGICA Consulting, Arthez-d’Asson, France; ^2^ Department of Neurology, Technical University of Munich, Klinikum rechts der Isar, Munich and Munich Cluster for Systems Neurology (SyNergy), Munich, Germany; ^3^ Staburo GmbH, Munich, Germany; ^4^ Polpharma Biologics S.A., Gdansk, Poland; ^5^ Hexal AG (a Sandoz company), Holzkirchen, Germany

**Keywords:** natalizumab, multiple sclerosis, neutralizing antibodies, anti-drug antibodies, immunology, biologic products, biosimilar, immunogenicity

## Abstract

**Background:**

Biosimilar natalizumab (biosim-NTZ) is the first biosimilar monoclonal antibody of reference natalizumab (ref-NTZ) for treatment of relapsing forms of multiple sclerosis (MS). Within the totality of evidence for demonstration of biosimilarity, immunogenicity assessments were performed in healthy subjects and patients with relapsing-remitting MS (RRMS) to confirm a matching immunogenicity profile between biosim-NTZ and ref-NTZ.

**Methods:**

Immunogenicity of biosim-NTZ versus ref-NTZ was evaluated in two pivotal clinical studies. In a comparative efficacy and safety study, patients with RRMS (n=264) received monthly infusions of biosim-NTZ/EU-ref-NTZ over 48 weeks. The primary endpoint period was Week 0 to Week 24. In a separate, comparative pharmacokinetic/pharmacodynamic (PK/PD) study, healthy subjects (n=450) received a single dose of biosim-NTZ, US-ref-NTZ or EU-ref-NTZ prior to an 85-day follow-up. In both studies, state-of-the-art, highly sensitive and drug tolerant bioanalytical assays were used to identify the proportion of participants with anti-drug antibodies (ADA) and neutralizing antibodies (NAb) against natalizumab over time.

**Results:**

In the comparative efficacy and safety study, biosim-NTZ and EU-ref-NTZ demonstrated similar incidences of overall ADA (79.4% vs 73.7%, respectively) and NAb (68.7% vs 66.2%, respectively) at Week 24. ADA titers over time were also concordant throughout the study period. Switching treatment from EU-ref-NTZ to biosim-NTZ had no impact on treatment-related ADA/NAb or clinical responses. Likewise, the single-dose PK/PD study reported matching overall incidence of ADA between treatment groups and matching ADA titer profiles over time.

**Conclusion:**

The immunogenicity profile of biosim-NTZ was confirmed to match that of ref-NTZ in healthy subjects and patients with RRMS by applying highly sensitive methods.

## 1 Introduction

The treatment landscape for multiple sclerosis (MS) was transformed over 20 years ago with the introduction of high-efficacy biologic disease modifying therapies (DMTs). In recent times, as these reference biologic medicines begin to lose market exclusivity, biosimilars to branded reference biologics are poised to enter the market, offering more cost-effective treatment options and expanding access to high-efficacy DMTs in MS (1, 2).

Therapeutic proteins, peptides and other biologic medicines have the potential to trigger an immunogenicity reaction, where the body produces antibodies against the medicine once exposed. Immunogenicity reactions may be associated with hypersensitivity reactions and reduced efficacy, resulting in treatment discontinuation (3). There may be concern that biosimilar medicines carry an increased immunogenicity risk compared with their reference medicine due to differences in the manufacturing process and/or product formulation. However, these concerns have not been substantiated in clinical practice and evidence indicates that biosimilar medicines offer continuity of outcomes, including absence of clinically meaningful differences in immunogenicity (4, 5).

Compared to the objective of reference medicine development programs, which is to establish the clinical benefit versus risk profile of the medicine based on *de novo* evidence for one or more therapeutic indications, the biosimilar development program is designed to provide the most sensitive head-to-head comparisons to detect any potential impact of observed differences in product quality attributes on known clinical outcomes. Accordingly, all biosimilar medicines approved in the US and Europe are required to demonstrate biosimilarity via a comprehensive ‘totality of evidence’ data package comprising extensive analytical, pharmacokinetic/pharmacodynamic (PK/PD), and clinical efficacy and safety data, as well as comprehensive assessments of immunogenicity to rule out any clinically meaningful differences of the biosimilar to the reference medicine (6–9).

Investigations into immunogenicity are weaved into the clinical components of a biosimilar development program to provide a comprehensive overview of a given biosimilar’s immunogenicity profile compared with its reference medicine. These investigations include incidence and magnitude of anti-drug antibody (ADA) responses that may affect the safety and/or effectiveness of any medicine by altering PK or promoting development of neutralizing antibodies (NAb) against the medicine (6–11).

The US Food and Drug Administration (FDA) and European Medicines Agency (EMA), as well as other international health authorities, recommend using a tiered strategy for immunogenicity sample testing with sequential screening and confirmatory assays, followed by semi-quantitation and characterization of ADA in terms of titer assessment and evaluation of neutralizing capacity (6, 7, 10, 11). A one-assay approach for the detection of ADA directed to either biosimilar or reference medicine has been recommended for comparative clinical studies to minimize a confounding influence of inter-assay variability (7, 12, 13). In many cases, the contemporary ADA and NAb assay methods applied for analysis of samples in biosimilar clinical studies have higher sensitivity and drug tolerance compared with those applied to support authorization of the reference medicine. These more sensitive assays typically reveal a higher incidence of treatment-emergent ADA than reported for earlier studies of the reference medicine (3, 13–15).

Biosimilar natalizumab (PB006; Polpharma Biologics S.A, marketed as Tyruko^®^, Sandoz [biosim-NTZ]) was approved by the FDA and EMA in 2023 and is the first biosimilar monoclonal antibody to reference natalizumab (Tysabri®; Biogen, Cambridge, MA, USA [ref-NTZ]) for relapsing forms of MS (16, 17). As an established treatment, extensive evidence has been collated for ref-NTZ over time, and it is known that natalizumab has identifiable, intrinsic T-dependent, immunogenic potential associated with the primary amino acid sequence of the variable fragment heavy-chain and light-chain domains (18–20). Previous studies of ref-NTZ have also reported that ADA against natalizumab can develop early during therapy (3, 21). The presence of ADA has been correlated with a reduction in serum natalizumab levels, and persistent antibody positivity has been associated with a reduction in treatment effectiveness in both clinical studies and real-world investigations (3, 18, 21). Furthermore, high ADA titers or persistent antibody positivity have been associated with infusion-related reactions (IRRs), including hypersensitivity reactions (3, 18, 21).

Herein, we provide details of the highly sensitive immunogenicity assessments used for comparison of biosim-NTZ versus ref-NTZ in the PB006 clinical development program, and report results from the comparative efficacy/safety and PK/PD studies that demonstrated matching immunogenicity profiles between biosim-NTZ and its reference medicine.

## 2 Materials and methods

### 2.1 Clinical studies

The immunogenicity of biosim-NTZ was compared to ref-NTZ in two randomized, comparative, pivotal clinical studies within the clinical development program of biosim-NTZ: the Antelope study in patients with relapsing-remitting MS (RRMS) (NCT04115488) (22), and a PK/PD study in healthy subjects (EudraCT: 2019-003874-15) (23). In each study, the immunogenicity sampling schedule was aligned with efficacy and safety monitoring or PK/PD sampling, respectively, to enable assessment of impact of any differences in magnitude of treatment-emergent ADA/NAb on overall clinical risk versus benefit.

#### 2.1.1 Antelope comparative efficacy and safety study in patients with RRMS

**Figure 1:**
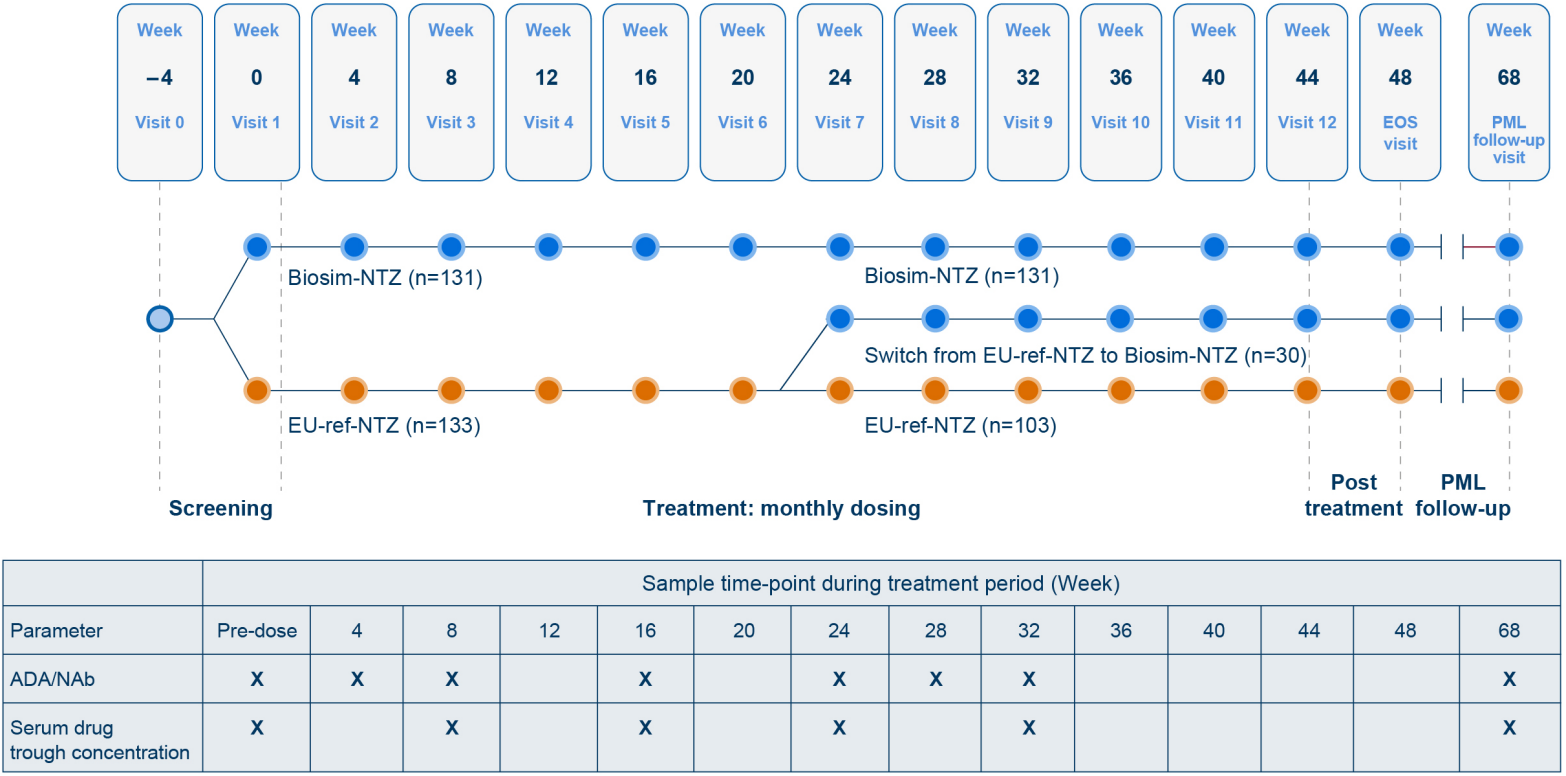
Antelope study design and immunogenicity assessment schedule (Safety Set). Blue circles signify contact points for patients receiving biosim-NTZ. Orange circles signify contact points for patients receiving EU-ref-NTZ. Hemmer B, et al. (22). Reprinted by permission of JAMA Network. ADA, anti-drug antibody; biosim-NTZ, biosimilar natalizumab; EOS, end of study; NAb, neutralizing antibody; PML, progressive multifocal leukoencephalopathy; ref-NTZ, reference natalizumab.

Antelope was a multicenter, double-blind, active-controlled, randomized, parallel-group study to assess similarity in efficacy, safety and immunogenicity of prolonged treatment with biosim-NTZ and EU-approved ref-NTZ (EU-ref-NTZ) in patients with RRMS (22). Eligible patients were randomly assigned (1:1) to receive intravenous infusions every 4 weeks of 300 mg biosim-NTZ or EU-ref-NTZ at a dose of 300 mg over 12 visits, for a total of 12 infusions. At treatment Week 24, the EU-ref-NTZ group was re-randomized and 30 patients were switched to continue treatment for 24 weeks with biosim-NTZ. The study design and bioanalytical sampling scheme for the Antelope study are summarized in **Figure 1**. The full details for this study, including clinical and safety methodology, have been reported elsewhere (22).

All patients treated with study drug were assessed as part of the Safety Set (n=264). Blood samples were collected to identify the proportion of patients with positive (transient and persistent) ADA and NAb against natalizumab, as well as any correlation between ADA status and clinical parameters (serum natalizumab concentration, efficacy, and safety), at each sampling visit. For example, published literature has identified an association between ADA formation and a reduction in serum drug concentration (3, 21). The relationship of hypersensitivity reactions, including IRRs, and symptoms corresponding to the Medical Dictionary for Regulatory Activities (MedDRA) term ‘anaphylaxis’ by ADA-positive/negative status and coincident ADA titer were also evaluated for all treatment groups.

#### 2.1.2 Comparative PK/PD study in healthy subjects

**Figure 2:**
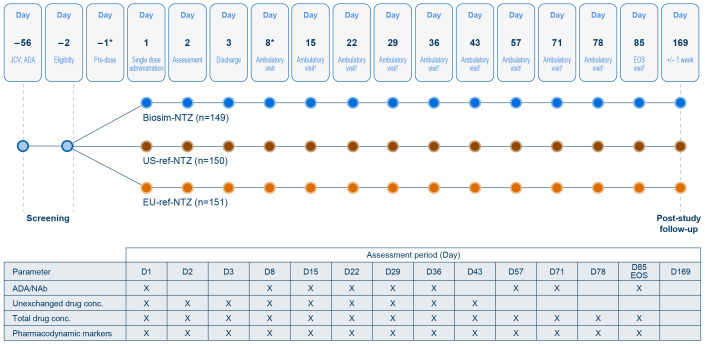
PK/PD study design and immunogenicity assessment schedule (Safety set). Dark blue circles signify contact points for patients receiving biosim-NTZ. Brown and orange circles signify contact points for patients receiving US- and EU-ref-NTZ, respectively. *Subjects were admitted to the studio center on Day –1 and discharged on Day 3 with subsequent ambulatory visits from Day 5. ^†^PK/PD sampling was performed via blood sampling, once pre-infusion and then at each ambulatory visit over 85 days post-infusion. Wessels H, et al. (23). Copyright © 2023 Polpharma Biologics S.A. reprinted by permission of Informa UK Limited, trading as Taylor & Francis Group https://www.tandfonline.com. ADA, anti-drug antibody; biosim-NTZ, biosimilar natalizumab; EOS, end of study; JCV, John Cunningham virus; NAb, neutralizing antibody; PD, pharmacodynamic; PK, pharmacokinetic; ref-NTZ, reference natalizumab.

This was a single-dose, randomized, double-blind, parallel group study to demonstrate similarity between biosim-NTZ and US-licensed ref-NTZ (US-ref-NTZ) or EU-ref-NTZ in terms of PK/PD in healthy subjects (N=453). The full study details for this study have been reported elsewhere (23). Subjects were randomized (1:1:1) based on body weight to receive a 1-hour, 3 mg/kg infusion of biosim-NTZ, US- or EU-ref-NTZ. PK endpoints included area under the curve from time of dosing extrapolated to infinity (AUC_0-inf_) and maximal concentration (C_max_) for total and unexchanged natalizumab, and PD endpoints included blood CD19+ cells, blood CD34+ cells, soluble mucosal vascular addressin cell adhesion molecule-1 (VCAM-1), and soluble molecular mucosal vascular addressin cell adhesion molecule-1 (MAdCAM-1) parameters. The study design and bioanalytical sampling scheme for the PK/PD study are summarized in **Figure 2**. All subjects treated with study drug were assessed as part of the Safety Set (n=450). Safety and immunogenicity were monitored throughout the study by repeated clinical and laboratory evaluations.

### 2.2 Immunogenicity assay test scheme

**Figure 3:**
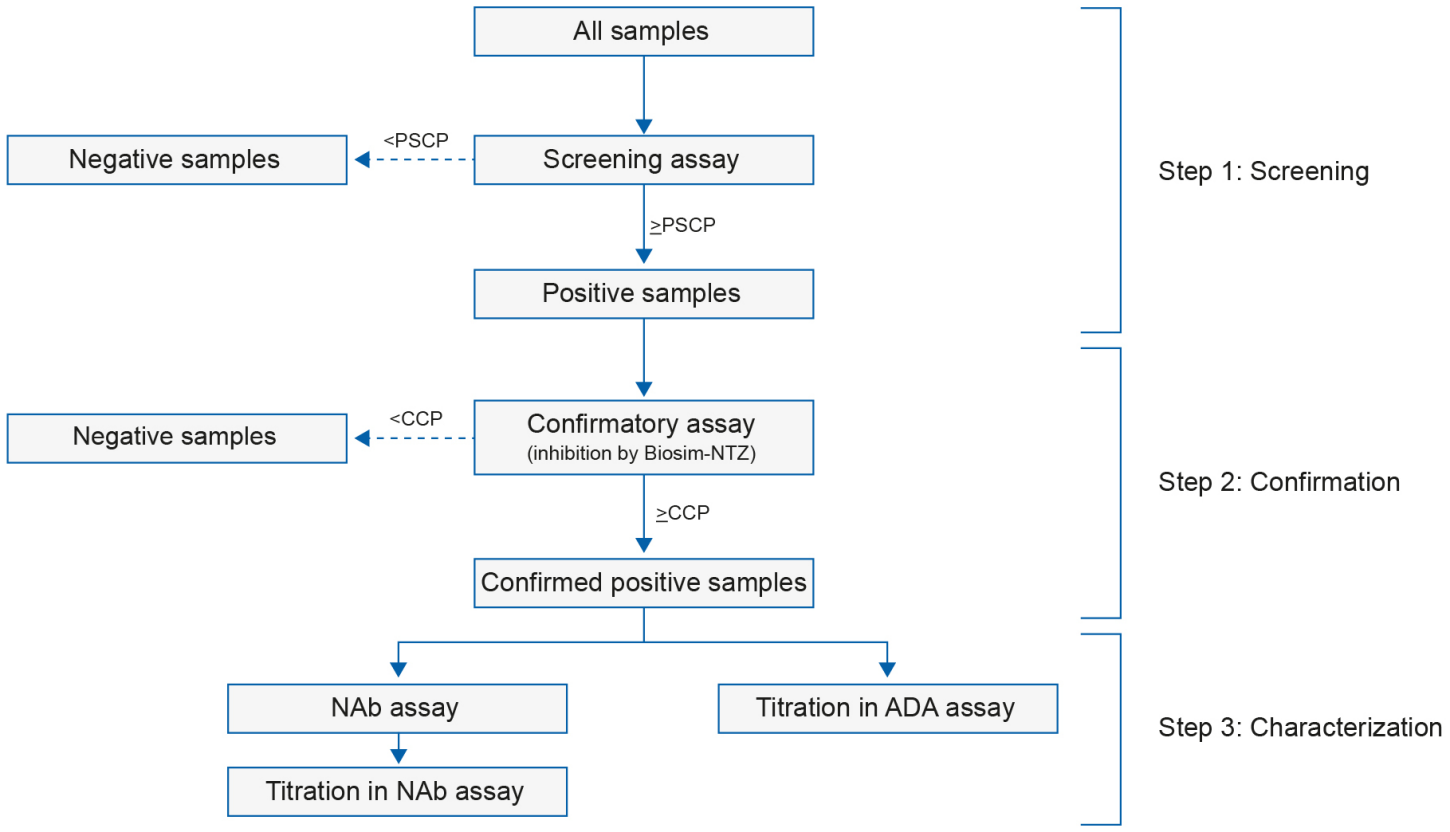
Immunogenicity assay scheme applied for monitoring ADA/NAb immune response. Clinical samples were tested for immunogenicity using a tiered, one-assay strategy for the detection of ADA and NAb as recommended by health authorities to minimize a confounding influence of inter-assay variability. Samples sequentially passed through screening and confirmatory assays, followed by semi-quantitation and characterization of ADA and NAb in terms of titer assessment and evaluation of neutralizing capacity. ADA, anti-drug antibody; biosim-NTZ, biosimilar natalizumab; CCP, confirmatory cut point; NAb, neutralizing antibody; PSCP, plate-specific cut point.

Novel, highly sensitive and drug-tolerant ADA assays were used to detect and to estimate titers of ADA-positive samples. The ADA titer corresponds to the reciprocal of highest dilution of sample yielding a signal above the titration assay threshold level (cut-point), and should reflect the final dilution of the test sample in the assay plate. The ADA/NAb test scheme applied for analysis of clinical samples across all studies is summarized in **Figure 3**. This generated a comprehensive data set to characterize the dynamics of the treatment-emergent ADA/NAb immune response to biosim-NTZ versus ref-NTZ.

#### 2.2.1 Electrochemiluminescence assay (ECLIA) for detection of ADA

**Figure 4:**
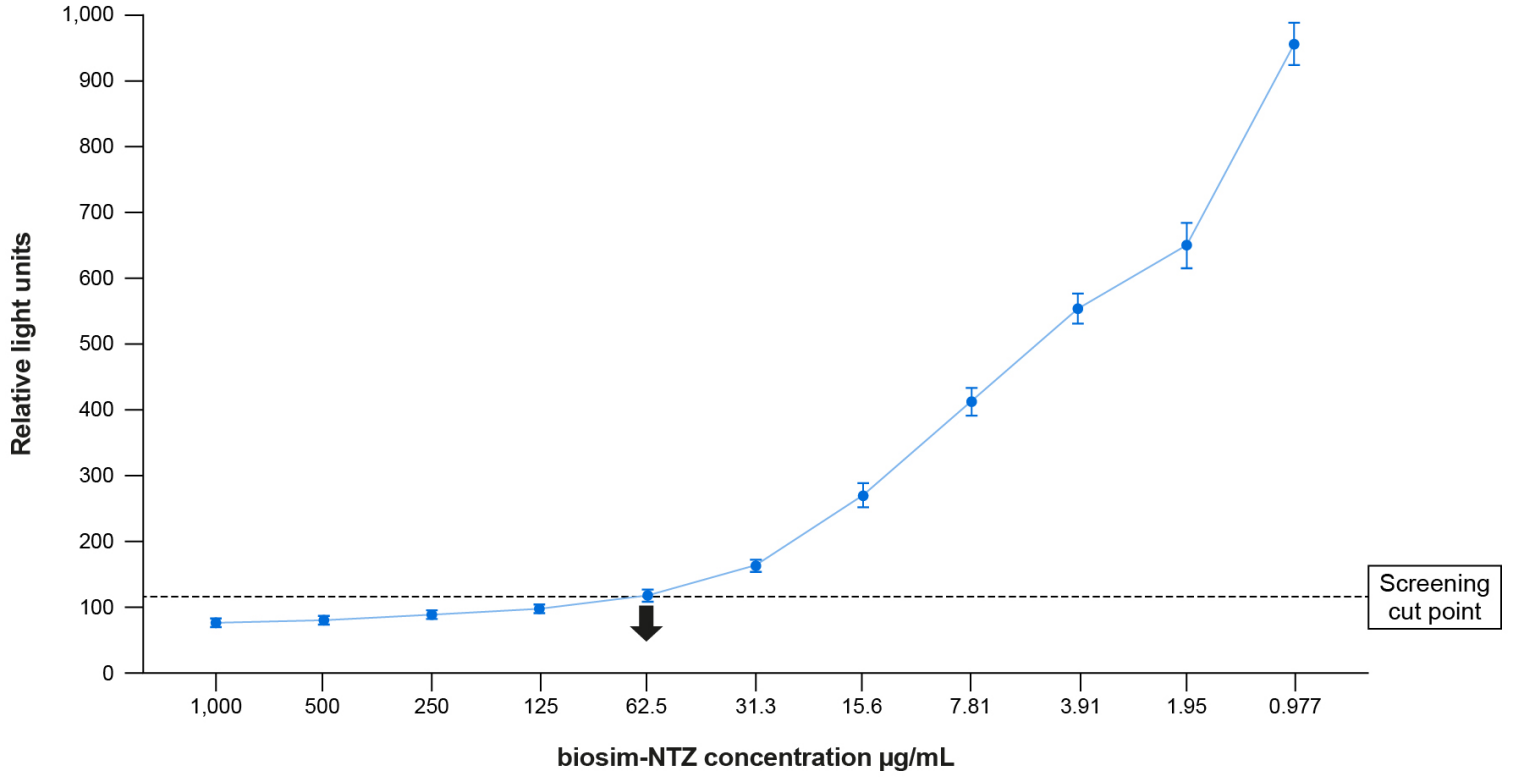
Drug tolerance of validated ADA assay method: 100 ng/mL positive control. The screening cut-point was calculated by multiplying the mean negative control signal for the assay run by a normalization factor of 1.30 representing the 95^th^ percentile of the distribution of log-transformed signals for 50 individual samples of human serum. ADA, anti-drug antibody; biosim-NTZ, biosimilar natalizumab.

Immunogenicity assays to detect binding ADA (screening and confirmatory assays) were based on an ECLIA platform. The suitability of the screening and confirmatory assay to detect anti-biosim-NTZ and anti-ref-NTZ antibodies in the same manner was demonstrated during method validation. In line with health authority guidance (10), the false positive rate for the screening and confirmatory assay was set to 5% and 1%, respectively. The applied minimal required dilution (MRD) of the assays was 1:10. The MRD in combination with acid pre-treatment of samples resulted in a relatively high sensitivity (3.88 ng/mL) and high drug tolerance (**Table 1**; **Figure 4**) of the method, which ensured the detection of ADA even with low magnitudes throughout the course of the clinical studies. To demonstrate the difference in sensitivity requirements for a biosimilar development program, the technical specifications of the assays used for the registrational biosim-NTZ and ref-NTZ immunogenicity analyses are shown in **Table 1**.

**Table 1:** Relative ADA assay tolerances for biosim-NTZ and ref-NTZ development studies.

Variable	Biosim-NTZ ADA assay	Ref-NTZ ADA assay (20, 29)
Assay	ECLIA with acid-dissociation sample pre-treatment	ELISA
Surrogate positive control antibody	Minipig anti-biosim-NTZ affinity-purified polyclonal antibody	Murine anti-ref-NTZ monoclonal antibody, 12C4
Sensitivity (LOD)	3.88 ng/mL	500 ng/mL
Drug tolerance	Drug/positive control ratio = 625	Drug/positive control ratio ≈ 2

ADA, anti-drug antibody; biosim-NTZ, biosimilar natalizumab; ECLIA, electrochemiluminescence immunoassay; ELISA, enzyme-linked immunosorbent assay; LOD, limit of detection; ref-NTZ, reference natalizumab.

The use of different positive control antibody reagents will bias comparison of reported assay sensitivity and drug tolerance.

If a sample tested positive in the screening assay (i.e. above the screening threshold level), the specificity of the signal to anti-natalizumab antibodies was tested in the confirmatory assay by spiking excess drug. The percent inhibition of the signals obtained when comparing unspiked and drug-spiked samples were required to be above the confirmatory threshold level to report a sample as ADA-positive. Samples below the screening or confirmatory threshold level were reported as ADA-negative. Confirmed positive samples were further tested for their magnitude in a dedicated titration assay (similar to the screening assay) by applying two-fold serial dilution steps in a negative control serum pool. The reported titer value represents the dilution which is required to cross the titer threshold level multiplied by the MRD.

ADA positivity was calculated as the percentage of participants with at least one treatment-emergent ADA-positive time-point, where the denominator was the number of ADA evaluable participants in the treatment group.

ADA positivity was concluded when at least one positive result was recorded in any post-baseline sample. Pre-existing ADA prevalence was determined by the proportion of participants testing positive for ADA at baseline. Transient positivity was defined as a patient with confirmed positive ADA in one or more sample(s) at non-consecutive post-dose visits, whereas persistent positivity was reported when confirmed positive antibodies were recorded in two or more consecutive positive samples at post-dose visits (with ≥16 weeks between first and last positive sample).

#### 2.2.2 NAb assay (cell-based assay)

Characterization of confirmed ADA-positive samples was tested in a dedicated cell-based NAb assay by flow cytometric detection of competitive inhibition of natalizumab binding to α4-integrin. Because the cognate antigen of natalizumab is the α4-integrin molecule expressed on the cell surface, the assay to detect NAb was configured to measure capacity of test samples containing anti-natalizumab antibodies to inhibit the binding of unlabeled natalizumab (EU-ref-NTZ) to α4-integrin expressed on Karpas 299 cells (CD45+ lymphocytes derived from a human T-cell lymphoma). Bound natalizumab was then detected using an anti-human IgG4-phycoerythrin antibody to generate a fluorescence signal measured in a flow cytometer; the magnitude of the fluorescence signal is reduced in the presence of anti-natalizumab antibodies that block binding to α4-integrin. Samples were pre-treated in a mild-acid dissociation step in combination with solid-phase extraction of the anti-natalizumab antibodies to improve drug tolerance. The sensitivity of the NAb assay was 205 ng/mL in the absence of natalizumab; 1.4 μg/mL positive control was detected in presence of up to 16 μg natalizumab/mL.

### 2.3 Statistical analysis

All statistical calculations were carried out using SAS language and procedures (SAS^®^ version 9.4, SAS Institute Inc., Cary, North Carolina, USA). Descriptive statistics were used to analyze the data. Ninety-five percent confidence intervals (CIs) of frequencies were calculated on the normal approximation. Box plots were employed for visual representation.

## 3 Results

### 3.1 Baseline characteristics

In the Antelope study, of 264 patients with RRMS, 131 (49.6%; mean age, 36.8 ± 9.1 years) received biosim-NTZ and 133 (50.4%; mean age, 36.6 ± 9.7 years) were treated with EU-ref-NTZ over 48 weeks (22). In the PK/PD study, of 450 healthy subjects, 149 (33.1%; mean age, 31 ± 10 years) participants received a single dose of biosim-NTZ, 150 (33.3%; mean age, 31 ± 11 years) received US-ref-NTZ and 151 (33.6%; mean age, 31 ± 11 years) received EU-ref-NTZ (23). The baseline demographics were comparable between the treatment groups for both studies.

### 3.2 Proportions of patients with ADA and NAb (transient and persistent)

**Figure 5:**
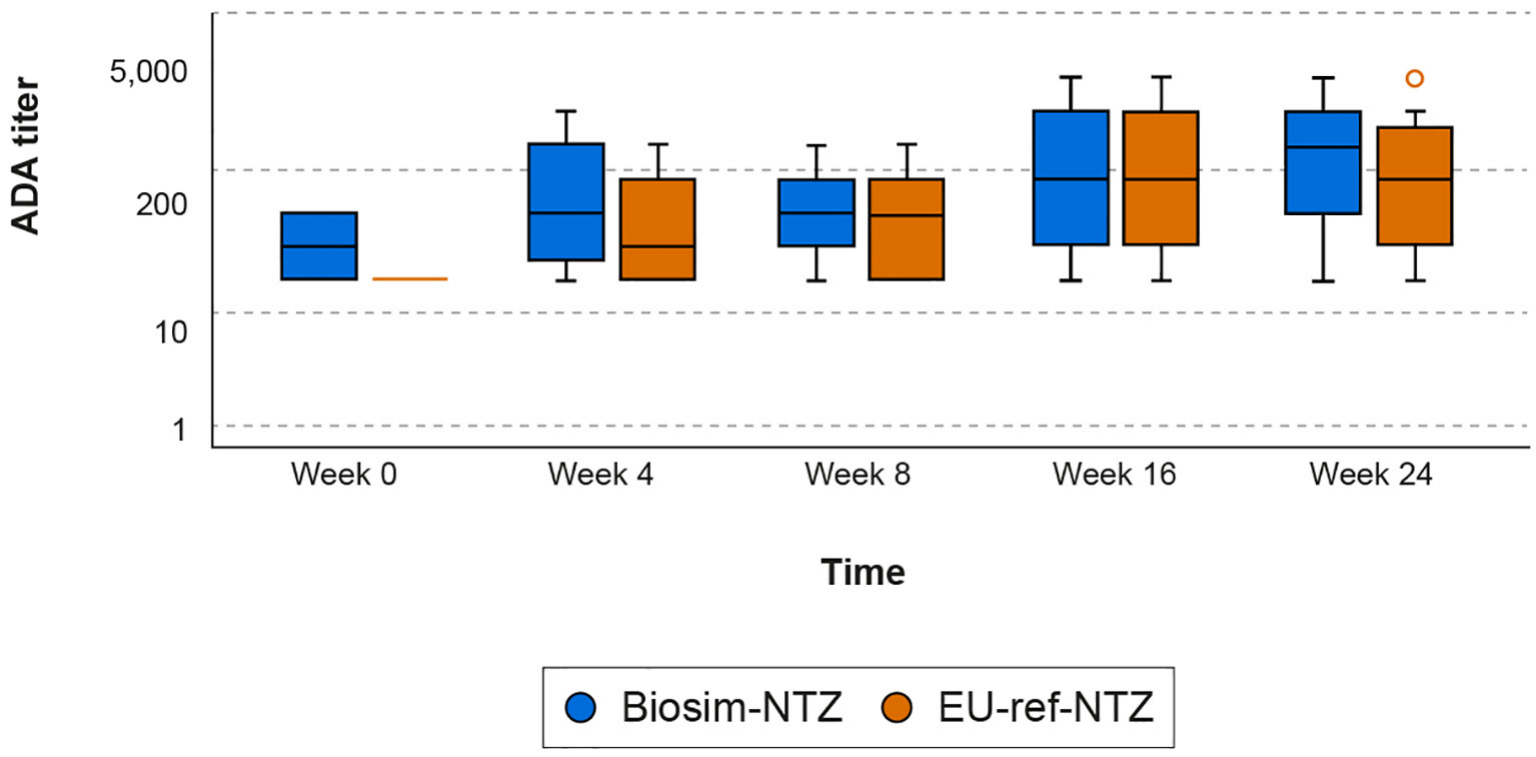
Box plot of ADA titers over time (Week 0–24) by treatment group (Antelope study). The ADA titer value represents the value of the highest dilution factor yielding a response greater than or equal to the cut point (threshold) multiplied by the MRD of 10; therefore, the minimum ADA titer value is 10. Descriptive statistics were used to analyze the data. Ninety-five percent confidence intervals of frequencies were calculated on the normal approximation. Boxes represent the Q1 (25^th^ percentile) to Q3 (75^th^ percentile) interquartile range, with the whiskers showing the minimum/maximum observations below/above the interquartile range; the horizontal line in each box represents the median value; the circles represent outlier values. ADA, anti-drug antibody; biosim-NTZ, biosimilar natalizumab; MRD, minimal required dilution; Q, quartile; ref-NTZ, reference natalizumab.

In patients with RRMS, concordance was observed between the biosim-NTZ and EU-ref-NTZ treatment groups in terms of overall ADA incidence (79.4% [95% CI: 72.5, 86.3] vs 73.7% [95% CI: 66.2, 81.2], respectively) and NAb incidence (68.7% [95% CI: 60.8, 76.6] vs 66.2% [95% CI: 58.1, 74.2], respectively) during the primary endpoint period of Week 0 to Week 24 (**Table 2**; **Figure 5**). ADA titer versus time profiles were also concordant across treatment groups throughout the study period of Week 0 to Week 48 (**Supplementary Table S1**).

**Table 2:** Summary of ADA and NAb response parameters at Week 24 and Week 48 (Antelope study).

Parameter	Week 24		Week 48	
	Biosim-NTZ (n=131)	EU-ref-NTZ (n=133)*	Biosim-NTZ (n=131)	EU-ref-NTZ (n=103)
Percentage ADA-positive				
Total treatment-emergent ADA, % (n)	79.4 (104)	73.7 (98)	79.4 (104)	73.8 (76)
Transient, % (n)	22.9 (30)	18.8 (25)	22.1 (29)	22.3 (23)
Persistent, % (n)	56.5 (74)	54.9 (73)	57.3 (75)	51.5 (53)
Geometric mean maximal ADA titer for total treatment-emergent ADA-positive samples (n)	223.5 (104)	150.7 (98)	229.1 (104)	131.5 (76)
Percentage NAb-positive, % (n)	68.7 (90)	66.1 (88)	68.7 (90)	67.0 (69)
Geometric mean maximal NAb titer for total treatment-emergent NAb-positive samples (n)	39.2 (90)	32.6 (88)	39.8 (90)	26.5 (69)

*The EU-ref-NTZ group contains both continuous and switched patients at Week 24.

ADA, anti-drug antibody; biosim-NTZ, biosimilar natalizumab; NAb, neutralizing antibody; ref-NTZ, reference natalizumab.

These findings were supported by the single-dose PK/PD study, which reported no detectable differences in ADA or NAb response dynamics in the biosim-NTZ treatment group compared with US-ref-NTZ or EU-ref-NTZ. The overall incidence of treatment-emergent ADA (across Days 8, 15, 22, 29, 36, 57, 71, and 85) was similar across all three treatment groups; 87.2% (biosim-NTZ) versus 92.0% (US-ref-NTZ) versus 86.8% (EU-ref-NTZ). ADA titer profiles at any timepoint post-infusion showed high variability and were similar, i.e. within a 2-fold difference, across all treatment groups (**Supplementary Table S2**).

### 3.3 Serum natalizumab concentration

**Figure 6:**
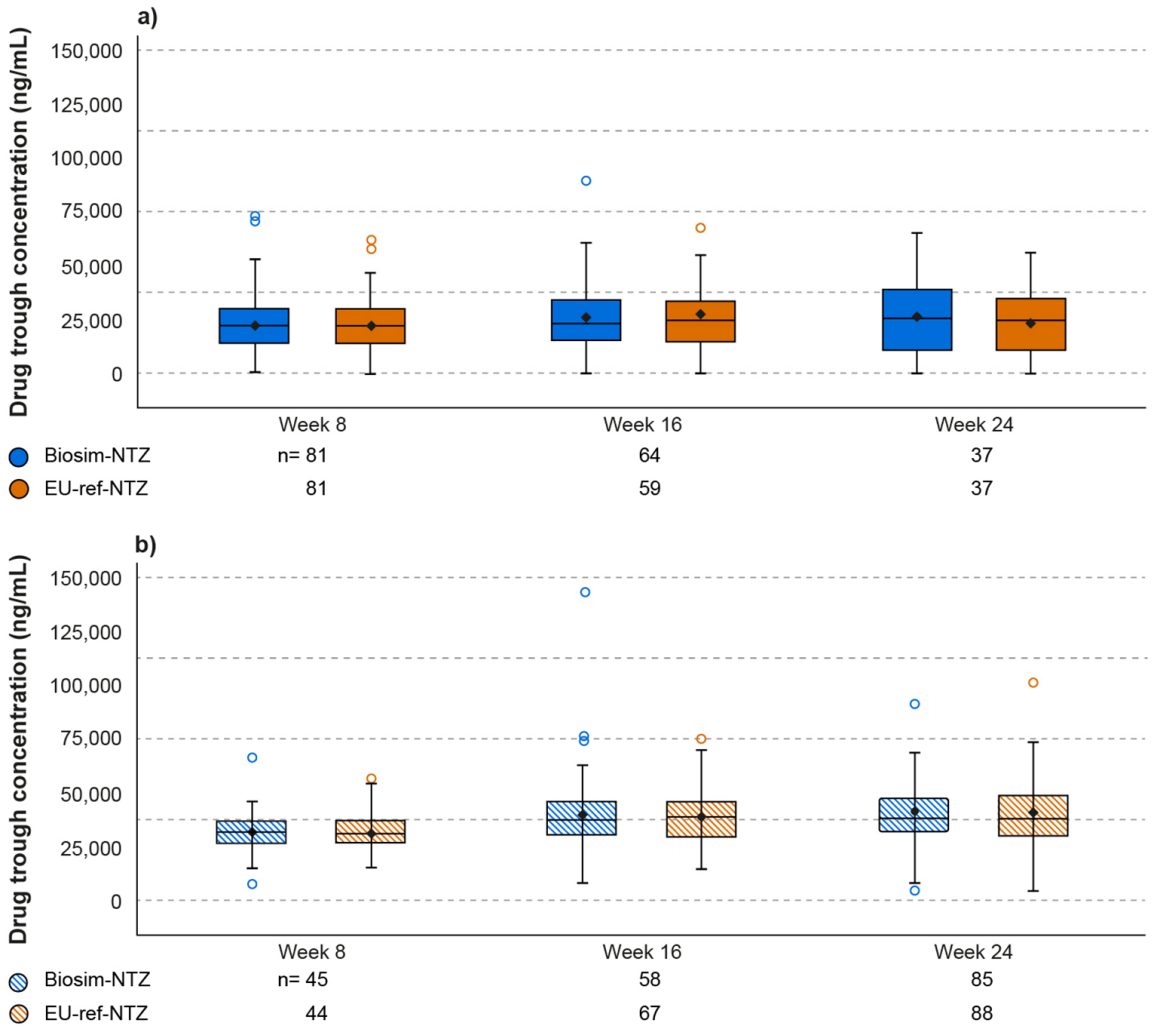
Serum natalizumab trough concentration over time (Week 8–24) by ADA category (Antelope study). **(A)** ADA-positive; **(B)** ADA-negative. Descriptive statistics were used to analyze the data. Ninety-five percent confidence intervals of frequencies were calculated on the normal approximation. Boxes represent the Q1 (25^th^ percentile) to Q3 (75^th^ percentile) interquartile range, with the whiskers showing the minimum/maximum observations below/above the interquartile range; the horizontal line in each box represents the median value; the circles represent outlier values. ADA, anti-drug antibody; biosim-NTZ, biosimilar natalizumab; ref-NTZ, reference natalizumab.

Throughout Antelope, the scale of reduction in serum total natalizumab trough concentration between biosim-NTZ and EU-ref-NTZ following treatment for 24 or 48 Weeks was concordant (**Table 3**; **Figure 6**). ADA-positive patients had lower trough concentrations compared with ADA-negative patients. NAb-positive patients had lower mean trough concentrations than ADA-positive patients. However, the extent of this decrease was equal across both treatment groups (data not shown).

**Table 3:** Serum total natalizumab trough concentration by ADA/NAb category at Week 24 (Antelope study).

Category	Geometric mean serum total natalizumab trough concentration (µg/mL) with 95% CIs			
	Biosim-NTZ (n=131)		EU-ref-NTZ (n=133)	
	n	Geometric mean (95% CI)	n	Geometric mean (95% CI)
ADA-negative	85	36,155.8 (32,705.1, 39,970.7)	88	36,200.9 (32,905.0, 39,826.8)
ADA-positive	37	11,375.8 (5,502.5, 23,518.3)	37	10,405.1 (5,208.2, 20,787.6)

ADA-negative: all patients who were negative for any timepoint between Week 0 to Week 24.

ADA-/NAb-positive: all patients with at least one positive timepoint between Week 0 to Week 24.

ADA, anti-drug antibody; biosim-NTZ, biosimilar natalizumab; CI, confidence interval; NAb, neutralizing antibody; ref-NTZ, reference natalizumab.

### 3.4 PK, PD and efficacy outcomes

Across the two clinical studies, both the dynamics of the treatment-emergent ADA/NAb immune response, as well as its impact on drug exposure, were comparable. In Antelope, no clinically meaningful differences in ADA/NAb profile or clinical measures of the cumulative number of new active lesions or annualized relapse rate were observed (**Table 4**).

**Table 4:** Summary of ADA and NAb response parameters versus clinical impact from Week 0 to Week 24 and Week 48 (Antelope study).

	Week 24		Week 48	
	Biosim-NTZ [n=131]	EU-ref-NTZ* [n=133]	Biosim-NTZ [n=131]	EU-ref-NTZ [n=103]
Cumulative number of new active lesions, n (SD)				
ADA-negative	1.8 (5.75) [n=27]	1.9 (3.53) [n=33]	1.9 (5.85) [n=26]	1.4 (2.27) [n=25]
ADA-positive	1.3 (2.80) [n=99]	1.9 (4.14) [n=94]	1.4 (2.93) [n=96]	2.7 (6.44) [n=71]
NAb-negative	1.1 (2.25) [n=13]	2.8 (3.55) [n=10]	1.1 (2.25) [n=13]	6.4 (11.62) [n=7]
NAb-positive	1.3 (2.89) [n=86]	1.8 (4.21) [n=84]	1.4 (3.03) [n=83]	2.3 (5.61) [n=64]
Annualized relapse rate				
ADA-negative	0.24 [n=27]	0.06 [n=35]	0.28 [n=27]	0.12 [n=27]
ADA-positive	0.20 [n=104]	0.18 [n=98]	0.14 [n=104]	0.14 [n=76]
NAb-negative	0.17 [n=14]	0.00 [n=10]	0.16 [n=14]	0.15 [n=7]
NAb-positive	0.20 [n=90]	0.21 [n=88]	0.14 [n=90]	0.13 [n=69]

*The EU-ref-NTZ group contains both continuous and switched patients at Week 24.

n = number of subjects with data available.

ADA, anti-drug antibody; biosim-NTZ, biosimilar natalizumab; NAb, neutralizing antibody; ref-NTZ, reference natalizumab; SD, standard deviation.

**Figure 7:**
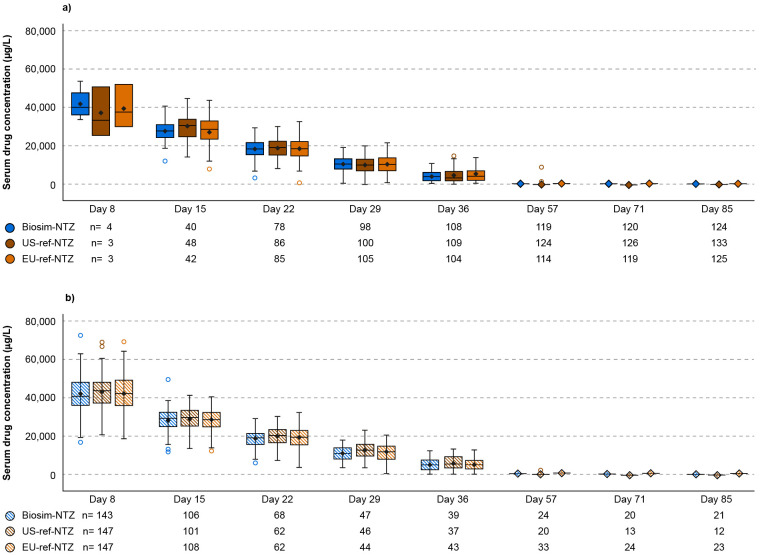
Box plot of serum drug concentration over time (Day 8–85) by ADA category (PK/PD study). **(A)** ADA-positive; **(B)** ADA-negative. Descriptive statistics were used to analyze the data. Ninety-five percent confidence intervals of frequencies were calculated on the normal approximation. n = number of subjects with data available. Boxes represent the Q1 (25^th^ percentile) to Q3 (75^th^ percentile) interquartile range, with the whiskers showing the minimum/maximum observations below/above the interquartile range; the horizontal line in each box represents the median value; the diamond symbol represents the mean value (geometric mean); the circles represent outlier values. ADA, anti-drug antibody; biosim-NTZ, biosimilar natalizumab; ref-NTZ, reference natalizumab.

Likewise, in the PK/PD study, no meaningful differences in the impact of ADA- or NAb-positivity on PK (AUC_0-inf_ and C_max_) or PD (blood CD19+ cells, blood CD34+ cells, VCAM-1, and MAdCAM-1) parameters were identified (**Supplementary Tables S3**, **S4**). Through to Day 85, serum drug concentration was similar across treatment groups when stratified by ADA status (**Figure 7**). ADA-positivity was not shown to affect percentage α4-integrin receptor saturation, which is a key parameter reflective of the mechanism of action of natalizumab, for any treatment group within the PK/PD study (**Supplementary Table S4**) (23).

### 3.5 Switching from EU-ref-NTZ to biosim-NTZ

Antelope demonstrated that switching from EU-ref-NTZ to biosim-NTZ had no impact on treatment-related ADA/NAb immune or clinical responses in patients with RRMS. In the switch arm (n=30) at Week 24 (prior to switch), 43.3% of patients were ADA-positive and 30.0% were NAb-positive. None of the patients who were ADA-negative or NAb-negative at Week 24 seroconverted following the switch from ref-NTZ to biosim-NTZ. ADA and NAb titers at Week 48 in the ADA-/NAb-positive patients did not increase from those at Week 24 in any patient switching from ref-NTZ to biosim-NTZ and was not associated with an enhanced anti-natalizumab ADA/NAb immune response in any patient (**Supplementary Table S5**).

### 3.6 Safety

The number of infusion-related reactions (IRRs) reported in the Antelope study was low. Out of 15 IRRs reported, ten IRRs were reported in the biosim-NTZ group (n=9) and five in the EU-ref-NTZ group (n=4). Of these, ten events (seven with biosim-NTZ and three with EU-ref-NTZ) were considered possibly or probably related to drug administration. Out of seven IRRs considered possibly or probably related to biosim-NTZ administration, five IRRs were detected in ADA-positive patients; one event of hypotension coincided with ADA positivity (high titer value of 40,960) and study discontinuation. In patients who switched from EU-ref-NTZ to biosim-NTZ at Week 24, treatment-related erythema and hypersensitivity reactions were reported at Day 169 (Week 24) in one patient who was ADA-negative at this time point, and was classified as persistently ADA-positive based on earlier timepoints (**Supplementary Table S6**). No hypersensitivity reactions were reported in the single-dose PK/PD study. There were no confirmed cases of progressive multifocal leukoencephalopathy (PML) reported in the Antelope study, which included a follow-up visit 24 weeks after the last dose of study drug to monitor for adverse events suggestive of PML (22).

## 4 Discussion

The immunogenicity of biosim-NTZ was evaluated directly in comparison to its reference medicine in two randomized, double-blind, parallel group, clinical studies. Highly sensitive and drug-tolerant ADA and NAb assays were developed and fully validated according to FDA and EMA guidelines (10, 24). Our clinical data demonstrated indistinguishable ADA/NAb response profiles for biosim-NTZ compared with ref-NTZ. In the therapeutic setting, both ADA and NAb incidence and ADA and NAb titers were similar between biosim-NTZ and EU-ref-NTZ over 48 weeks of repeated treatment, with no differences observed for drug trough concentration or ADA/NAb status on efficacy and safety. Incidence of ADA immune responses observed for biosim-NTZ versus EU-ref-NTZ was 79.4% vs 73.7% and for NAb, 68.7% vs 66.2%, respectively, in patients with RRMS. Switching from EU-ref-NTZ to biosim-NTZ did not impact immunogenicity or clinical outcomes. Consistent with the known effects of natalizumab, ADA-positive status resulted in reduced natalizumab serum concentrations; however, there was no difference in the scale of reductions for biosim-NTZ compared with EU-ref-NTZ. Likewise in the PK/PD study, ADA and NAb incidences of 87% vs 87–92% and 84% vs 77–87% were observed following single-dose administration in healthy subjects, respectively, concluding no apparent treatment-related differences in the impact of ADA-positive status on PK/PD outcomes. Furthermore, the number of hypersensitivity reactions reported in the Antelope study was similarly low across treatment groups with none reported in the PK/PD study in healthy subjects (22, 23). Biosim-NTZ and ref-NTZ share 100% amino acid sequence identity; accordingly, they are expected to have equivalent intrinsic immunogenic potential. Extrinsically, biosim-NTZ is expressed in a different cell line to the murine NS0 cell line used for ref-NTZ, and there are some detectable differences in post-translational glycosylation patterns. However, biosim-NTZ has lower levels of galactose-α-1,3-galactosylation, which reduces potential risk from the immunogenicity or antigenicity perspective. The cell culture process for biosim-NTZ is performed using a suspension-adapted Chinese hamster ovary cell line, compared with the murine myeloma cell line used in the manufacture of ref-NTZ (18). In principle, this difference could also reduce the biosimilar’s risk for immunogenicity associated with the higher levels of non-human glycans/glycosidic linkages, that are commonly observed for therapeutic proteins expressed in the NS0 cell line (25). Furthermore, matching analytical similarity for biosim-NTZ to ref-NTZ has been demonstrated for structural attributes associated with immunogenicity risk including molecular size variants, sub-visible particles, and stability under accelerated and stress conditions (26). The data presented here clinically support the analytical and functional characterization data, demonstrating that biosim-NTZ matches ref-NTZ in critical quality attributes pertaining to immunogenicity (22, 23).

A recent investigation into immunogenicity assessments of 22 FDA-approved biosimilar monoclonal antibodies reported a high level of consistency between the evaluated biosimilars and their reference medicines in terms of ADA/NAb incidence and ADA titer, which aligned with comparative impact on PK outcomes (27). The results of this analysis, as well as the matching immunogenicity profile demonstrated in the current studies between biosim-NTZ and ref-NTZ, further validate the totality of evidence approach to demonstrating biosimilarity, and confirm that no substantial differences in PK parameters as a result of ADA/NAb presence should be expected between a biosimilar medicine and its reference.

In the clinical studies discussed in this present publication, higher ADA and NAb incidences were detected compared to those previously reported for ref-NTZ (ADA incidence of 4.5%–14.1% in patients with RRMS) (3, 19, 21, 28). The most plausible explanation is the higher drug tolerance of the biosim-NTZ ADA and NAb assays achieved by inclusion of sample pre-treatment to reduce drug interference allied to the superior sensitivity of the ECLIA (**Table 1**). Effective sensitivity of the ADA assay applied to support marketing approval of ref-NTZ was limited by drug interference: at 100 ng 12C4 positive control antibody/mL serum, the drug tolerance limit was estimated to be only 40 ng natalizumab/mL serum (3, 29), thereby underestimating the ‘true’ ADA response because drug trough levels in the clinical setting are typically higher than this level. The US Prescribing Information for ref-NTZ states that “*the assays used were unable to detect low to moderate levels of antibodies to natalizumab*” (18) and it has been postulated that the reported ADA incidence for ref-NTZ underestimates the ADA incidence that would be detectable when applying a contemporary, highly drug-tolerant ADA assay (18, 20). Using a more sensitive assay, ADA have elsewhere been detected in 42/73 (58%) of tested ref-NTZ-treated patients with MS (3), which is considerably higher than that reported above for the originator studies (19, 21, 28). The EMA has stated that the validation approach for the historic ELISA applied to monitor ADA in clinical studies for ref-NTZ is not fully in line with current recommendations on assessment of immunogenicity (20). Moreover, given the aforementioned absence of standardized methodology and reference materials for ADA testing, comparison of different assay outputs must be approached with caution (13).

As discussed in Section 2.1.1, the immunogenicity-related safety of biosim-NTZ relative to EU-ref-NTZ was determined by comparison of incidence and severity of hypersensitivity reactions, including IRRs. Symptoms corresponding to the MedDRA term ‘anaphylaxis’ by ADA-positive/negative status and coincident ADA titer were also evaluated for all treatment groups and the frequency of these adverse events will be monitored by routine post-authorization pharmacovigilance. The weight-of-evidence from the comparative clinical studies indicated that biosim-NTZ has a similar immunogenicity profile to ref-NTZ using appropriately sensitive methodology. These data form part of the ‘totality of evidence’ data package for biosim-NTZ, which has been accepted for publication (26). There were no unexpected or new safety signals identified in the biosim-NTZ clinical studies, including incidence of PML (22, 23). Further, no cases of PML have been reported in the literature for biosim-NTZ in clinical practice to date. Ongoing evaluation of the incidence of PML will be monitored for biosim-NTZ by post-authorization pharmacovigilance (including a US Risk Evaluation and Mitigation Strategy and European Risk Management Program), noting that the prescribing information for all natalizumab product versions advises close monitoring for symptoms of PML (18, 19, 30, 31).

## 5 Conclusion

Using appropriately sensitive methodology as reported in the present publication, we have demonstrated that the immunogenicity profiles of biosim-NTZ and ref-NTZ are indistinguishable in healthy subjects and patients with RRMS in the tested setting. These findings confirm the biosimilarity of biosim-NTZ and ref-NTZ with no safety or efficacy concerns identified to be associated with immunogenicity.

## Data Availability

The raw data supporting the conclusions of this article for the PB006-03-01 Antelope study are disclosed on clinicaltrials.gov (NCT04115488), and within the EudraCT system (2019-003874-15) for the PB006-01-03 PK/PD study. Further inquiries can be directed to the corresponding author.
