# Supplementary material for: Comparative immunogenicity assessment of biosimilar natalizumab to its reference medicine: a matching immunogenicity profile
Source: Front Immunol. 2024 Dec 19;15:1414304. doi: 10.3389/fimmu.2024.1414304 (PMC11693714; doi:10.3389/fimmu.2024.1414304)
Supplement: Supplementary file 1 [file DataSheet1.docx]

Supplementary Material

# Supplementary Tables

**Table 1. ADA/NAb responses over time (Week 0–48) by treatment group (Antelope study).**

|  |  |  | | | | | | |
| --- | --- | --- | --- | --- | --- | --- | --- | --- |
|  | **Pre-dose** | **Treatment week** | | | | | | |
|  |  | **4** | **8** | **16** | **24** | **28** | **32** | **48** |
| **Biosim-NTZ** | | | | | | | | |
| **Total subjects** | 131 | 127 | 127 | 123 | 122 | 119 | 119 | 117 |
| **ADA-positive, n (%)** | 9 (7) | 69 (54) | 82 (65) | 64 (52) | 37 (30) | 27 (23) | 27 (23) | 13 (11) |
| **ADA titer:**   - **Geometric mean** - **Median** - **Min–max** | 40.0  40.0  20–80 | 145.7  80.0  20–40,960 | 122.6  80.0  20–10,240 | 248.4  160.0  20–163,840 | 393.0  320.0  20–163,840 | 553.9  320.0  40–327,680 | 470.3  160.0  20–81,920 | 508.0  160.0  20–40,960 |
| **NAb-positive, n (%)** | 0 | 41 (32) | 80 (63) | 42 (34) | 25 (20) | 16 (13) | 17 (14) | 8 (7) |
| **EU-ref-NTZ** | | | | | | | | |
| **Total subjects** | 103 | 101 | 98 | 96 | 95 | 95 | 95 | 93 |
| **ADA-positive, n (%)** | 7 (7) | 50 (50) | 60 (61) | 41 (43) | 24 (25) | 21 (22) | 19 (20) | 9 (10) |
| **ADA titer:**   - **Geometric mean** - **Median** - **Min–max** | 20.0  20.0  20–20 | 69.6  40.0  20–5,120 | 67.8  40.0  20–10,240 | 176.7  160.0  20–163,840 | 165.4  160.0  20–327,680 | 285.1  120.0  20–655,360 | 287.6  160.0  20–327,680 | 570.2  160.0  20–655,360 |
| **NAb-positive, n (%)** | 0 | 26 (26) | 58 (59) | 27 (28) | 16 (17) | 8 (8) | 11 (12) | 4 (4) |
| **Switch from EU-ref-NTZ to biosim-NTZ at Week 24** | | | | | | | | |
| **Total subjects** | 30 | 30 | 30 | 30 | 30 | 29 | 28 | 29 |
| **ADA-positive, n (%)** | 1 (3) | 17 (57) | 21 (70) | 18 (60) | 13 (43) | 14 (48) | 9 (32) | 5 (17) |
| **ADA titer:**   - **Geometric mean** - **Median** - **Min–max** | 40.0  40.0  40–40 | 127.0  80.0  20–5,120 | 103.3  80.0  20–2,560 | 294.9  160.0  20–81,920 | 386.6  160.0  40–40,960 | 239.7  80.0  20–81,920 | 525.0  160.0  20–81,920 | 761.1  10,250  20–40,960 |
| **NAb-positive, n (%)** | 0 | 8 (27) | 19 (63) | 13 (43) | 9 (30) | 4 (14) | 5 (18) | 3 (10) |
|  |  |  |  |  |  |  |  |  |

ADA-/NAb-positive = all patients with at least one positive timepoint between Week 0 and Week 24. Early discontinuation visits with a study day ≤181 were assigned to Week 0–24.

ADA, anti-drug antibody; biosim-NTZ, biosimilar natalizumab; NAb, neutralizing antibody; ref-NTZ, reference natalizumab.

**Table 2. ADA titers during the 85-day assessment period by treatment group (PK/PD study).**

ADA, anti-drug antibody; biosim-NTZ, biosimilar natalizumab; NAb, neutralizing antibody; PK/PD, pharmacokinetic/pharmacodynamic; ref-NTZ, reference natalizumab.

|  |  |  | | | | | | | | |
| --- | --- | --- | --- | --- | --- | --- | --- | --- | --- | --- |
|  | **Pre-dose** | **Day post-single dose administration** | | | | | | | | |
|  |  | **8** | **15** | **22** | **29** | **36** | **57** | **71** | | **85** |
| **Biosim-NTZ (n=149)** | | | | | | | | | | |
| **Total subjects, n** | 149 | 147 | 146 | 146 | 145 | 147 | 144 | 140 | 145 | |
| **ADA-positive, n (%)** | 16 (11) | 4 (3) | 40 (27) | 78 (53) | 98 (68) | 108 (73) | 119 (83) | 120 (86) | 124 (86) | |
| **ADA titer:**   - **Geometric mean** - **Median** - **Min–max** | 40.0  30.0  20–160 | 20.0  20.0  20–20 | 48.6  40.0  20–1280 | 118.3  80.0  20–40,960 | 184.1  160.0  20–20,480 | 171.4  160.0  20–20,480 | 378.8  320.0  20–20,480 | 425.5  480.0  20–10,240 | 453.8  640.0  20–20,480 | |
| **NAb-positive, n (%)** | 0 | 0 | 6 (4) | 42 (29) | 72 (50) | 96 (65) | 116 (81) | 115 (82) | 118 (81) | |
| **EU-ref-NTZ (n=151)** | | | | | | | | | | |
| **Total subjects, n** | 151 | 150 | 150 | 148 | 149 | 148 | 147 | 143 | 148 | |
| **ADA-positive, n (%)** | 26 (17) | 3 (2) | 42 (28) | 85 (57) | 105 (70) | 104 (70) | 114 (78) | 119 (83) | 125 (84) | |
| **ADA titer:**   - **Geometric mean** - **Median** - **Min–max** | 42.0  20.0  20–2560 | 20.0  20.0  20–20 | 44.3  40.0  20–320 | 133.6  160.0  20–5,120 | 345.0  320.0  20–81,920 | 277.4  320.0  20–20,480 | 604.7  640.0  20–20,480 | 536.5  640.0  20–10,240 | 501.3  640.0  20–10,240 | |
| **NAb-positive, n (%)** | 0 | 0 | 1 (1) | 53 (36) | 83 (56) | 94 (64) | 112 (76) | 110 (77) | 115 (78) | |
| **US-ref-NTZ (n=150)** | | | | | | | | | | |
| **Total subjects, n** | 150 | 150 | 149 | 148 | 146 | 146 | 144 | 139 | 145 | |
| **ADA-positive, n (%)** | 22 (15) | 3 (2) | 48 (32) | 86 (58) | 100 (68) | 109 (75) | 124 (86) | 126 (91) | 133 (92) | |
| **ADA titer:**   - **Geometric mean** - **Median** - **Min–max** | 36.2  40.0  20–160 | 20.0  20.0  20–20 | 58.2  40.0  20–1280 | 108.5  80.0  20–20,480 | 234.0  160.0  20–81,920 | 218.4  160.0  20–40,960 | 495.0  640.0  20–20,480 | 555.8  640.0  20–20,480 | 554.7  640.0  20–20,480 | |
| **NAb-positive, n (%)** | 0 | 0 | 5 (3) | 57 (39) | 79 (54) | 93 (64) | 119 (83) | 119 (86) | 123 (85) | |
|  |  |  |  |  |  |  |  |  |  | |
|  |  |  |  |  |  |  |  |  |  | |

**Table 3. Summary of PK parameters by ADA/NAb category (PK/PD study).**

ADA, anti-drug antibody; AUC_0-inf_, area under the curve from time of dosing extrapolated to infinity; biosim-NTZ, biosimilar natalizumab; CI, confidence interval; C_max_, maximum concentration; GMR, geometric mean ratio; NAb, neutralizing antibody; PD, pharmacodynamics; PK, pharmacokinetics; ref-NTZ, reference natalizumab.

|  |  | | |
| --- | --- | --- | --- |
| **PK parameter** | **Pairwise comparison** | | |
|  | **Test (n) versus Reference (n)** | **GMR** | **90% CI** |
| **AUC_0-inf_ (h*mg/L)** | | | |
| **ADA-positive** | Biosim-NTZ (125) vs EU-ref-NTZ (128) | 98.72 | 93.89, 103.79 |
|  | Biosim-NTZ (125) vs US-ref-NTZ (137) | 95.80 | 91.19, 100.64 |
|  | EU-ref-NTZ (128) vs US-ref-NTZ (137) | 97.04 | 92.40, 101.91 |
| **NAb-positive** | Biosim-NTZ (120) vs EU-ref-NTZ (115) | 99.73 | 94.80, 104.91 |
|  | Biosim-NTZ (120) vs US-ref-NTZ (131) | 96.87 | 92.23, 101.75 |
|  | EU-ref-NTZ (115) vs US-ref-NTZ (131) | 97.14 | 92.43, 102.08 |
| **C_max_ (h*mg/L)** | | | |
| **ADA-positive** | Biosim-NTZ (125) vs EU-ref-NTZ (128) | 96.46 | 92.66, 100.41 |
|  | Biosim-NTZ (125) vs US-ref-NTZ (137) | 97.20 | 93.44, 101.12 |
|  | EU-ref-NTZ (128) vs US-ref-NTZ (137) | 100.78 | 96.90, 104.81 |
| **NAb-positive** | Biosim-NTZ (120) vs EU-ref-NTZ (115) | 98.04 | 94.19, 102.04 |
|  | Biosim-NTZ (120) vs US-ref-NTZ (131) | 98.28 | 94.55, 102.16 |
|  | EU-ref-NTZ (115) vs US-ref-NTZ (131) | 100.25 | 96.40, 104.25 |
|  |  |  |  |

**Table 4. Summary of PD parameters by ADA/NAb category (PK/PD study).**

ADA, anti-drug antibody; biosim-NTZ, biosimilar natalizumab; CI, confidence interval; GMR, geometric mean ratio; NAb, neutralizing antibody; PD, pharmacodynamics; PK, pharmacokinetics; ref-NTZ, reference natalizumab.

|  |  | | |
| --- | --- | --- | --- |
| **PD parameter** | **Pairwise comparison** | | |
|  | **Test (n) vs Reference (n)** | **GMR** | **90% CI** |
| **AUEC_0-12w_ α4-integrin receptor saturation (%*h)** | | | |
| **ADA-positive** | Biosim-NTZ (111) vs EU-ref-NTZ (120) | 101.56 | 97.36, 105.93 |
|  | Biosim-NTZ (111) vs US-ref-NTZ (117) | 96.98 | 92.95, 101.18 |
|  | EU-ref-NTZ (120) vs US-ref-NTZ (117) | 95.49 | 91.60, 99.55 |
| **NAb-positive** | Biosim-NTZ (108) vs EU-ref-NTZ (110) | 102.41 | 98.04, 106.97 |
|  | Biosim-NTZ (108) vs US-ref-NTZ (111) | 97.34 | 93.20, 101.67 |
|  | EU-ref-NTZ (110) vs US-ref-NTZ (111) | 95.05 | 91.03, 99.25 |
| **AUEC_4-12w_ α4-integrin receptor saturation (%*h)** | | | |
| **ADA-positive** | Biosim-NTZ (121) vs EU-ref-NTZ (123) | 106.47 | 95.09, 119.20 |
|  | Biosim-NTZ (121) vs US-ref-NTZ (126) | 97.44 | 87.09, 109.02 |
|  | EU-ref-NTZ (123) vs US-ref-NTZ (126) | 91.52 | 81.83, 102.35 |
| **NAb-positive** | Biosim-NTZ (117) vs EU-ref-NTZ (113) | 109.18 | 97.05, 122.83 |
|  | Biosim-NTZ (117) vs US-ref-NTZ (120) | 98.44 | 87.65, 110.55 |
|  | EU-ref-NTZ (113) vs US-ref-NTZ (120) | 90.16 | 80.20, 101.36 |
| **AUEC_0-12w_ CD19+ (10^6^/L*h)** | | | |
| **ADA-positive** | Biosim-NTZ (111) vs EU-ref-NTZ (120) | 91.64 | 80.37, 104.50 |
|  | Biosim-NTZ (111) vs US-ref-NTZ (117) | 91.32 | 79.71, 104.61 |
|  | EU-ref-NTZ (120) vs US-ref-NTZ (117) | 99.65 | 87.45, 113.54 |
| **NAb-positive** | Biosim-NTZ (108) vs EU-ref-NTZ (110) | 93.75 | 81.93, 107.27 |
|  | Biosim-NTZ (108) vs US-ref-NTZ (111) | 93.08 | 81.35, 106.51 |
|  | EU-ref-NTZ (110) vs US-ref-NTZ (111) | 99.29 | 86.91, 113.43 |
|  |  |  |  |

**Table 5. ADA and NAb outcomes at Week 24 versus 48 for subjects who switched from EU-ref-NTZ to biosim-NTZ at Week 24 (Antelope study; Safety analysis Set [n=30]).**

ADA, anti-drug antibody; biosim-NTZ, biosimilar natalizumab; ED, early discontinuation; NAb, neutralizing antibody; NR, no result; ref-NTZ, reference natalizumab.

|  | | |  | | |
| --- | --- | --- | --- | --- | --- |
| **Week 24 (Pre-switch)** | | | **Week 48 (Post-switch)** | | |
| **ADA status** | **ADA titer** | **NAb status** | **ADA status** | **ADA titer** | **NAb status** |
| Negative |  | Negative | Negative |  | Negative |
| Positive | 40 | Positive | Negative |  | Negative |
| Positive | 1,280 | Positive | Negative |  | Negative |
| Negative |  | Negative | ED |  |  |
| Negative |  | Negative | NR |  |  |
| Negative |  | Negative | Negative |  | Negative |
| Positive | 320 | Positive | Positive | 20 | Negative |
| Positive | 40,960 | Positive | Positive | 40,960 | Positive |
| Positive | 160 | Positive | Negative |  | Negative |
| Negative |  | Negative | Negative |  | Negative |
| Positive | <20 | Negative | Positive | <20 | Negative |
| Negative |  | Negative | Negative |  | Negative |
| Negative |  | Negative | Negative |  | Negative |
| Negative |  | Negative | Negative |  | Negative |
| Positive | 160 | Positive | Negative |  | Negative |
| Negative |  | Negative | Negative |  | Negative |
| Negative |  | Negative | Negative |  | Negative |
| Negative |  | Negative | Negative |  | Negative |
| Positive | <20 | Negative | Negative |  | Negative |
| Negative |  | Negative | Negative |  | Negative |
| Positive | 80 | Negative | Negative |  | Negative |
| Positive | 40,960 | Positive | Positive | 20,480 | Positive |
| Negative |  | Negative | Negative |  | Negative |
| Negative |  | Negative | Negative |  | Negative |
| Negative |  | Negative | Negative |  | Negative |
| Positive | 160 | Positive | Negative |  | Negative |
| Positive | 40 | Positive | Positive | 20 | Positive |
| Negative |  | Negative | Negative |  | Negative |
| Negative |  | Negative | Negative |  | Negative |
| Positive | 80 | Negative | Negative |  | Negative |
|  |  |  |  |  |  |

**Table 6. Breakdown of patients with ≥1 hypersensitivity reaction (SMQ anaphylactic reaction or hypersensitivity) (Safety Analysis Set)**

*According to Common Terminology Criteria for Adverse Events. ^†^Classified as an adverse event of special interest.

Coincident ADA positive = confirmed ADA positive result in the sample taken just prior to dose associated with the TEAE.

ADA, anti-drug antibody; biosim-NTZ, biosimilar natalizumab; ref-NTZ, reference natalizumab; SMQ, Standardized MedDRA Query; TEAE, treatment-emergent adverse event.

|  |  |  |  |  |  |  |
| --- | --- | --- | --- | --- | --- | --- |
| **Patient** | **ADA status (Coincident ADA titer)** | **Start date** | **Preferred term** | **Causality** | **Severity*** | **Study completion** |
| **Biosim-NTZ (n=9)** | | | | | | |
| 1 | Negative | 234 | Allergic sinusitis | Unlikely related | Mild | Completed |
| 2 | Negative | 182 | Rhinitis allergic | Not related | Mild | Completed |
| 3 | Negative | 71 | Pruritus | Possibly related | Moderate | Discontinued due to severe itching of the whole body skin |
| 4 | Negative | 214 | Hypotension | Possibly related | Mild | Completed |
| 5 | Positive  (2,560) | 79 | Stomatitis | Not related | Mild | Completed |
| 6 | Positive  (5,120) | 29 | Pruritus | Possibly related | Mild | Discontinued due to itchy skin on the palms of the hand^†^ |
| 7 | Positive  (20,480) | 29 | Erythema | Possibly related | Mild | Discontinued due to urticaria^†^ |
|  | Positive  (10,240) | 85 | Urticaria | Probably related | Severe |  |
| 8 | Positive  (40,960 [at discontinuation]) | 310 | Hypotension | Possibly related | Moderate | Discontinued due to hypotension |
| 9 | Positive  (160) | 86 | Urticaria | Possibly related | Moderate | Discontinued due to acute urticaria^†^ |
| **EU-ref-NTZ (n=4)** | | | | | | |
| 1 | Positive  (40) | 99 | Rhinitis allergic | Not related | Moderate | Completed |
| 2 | Positive  (160 [at Week 16]) | - | Rash | Possibly related | Mild | Completed |
| 3 | Positive  (20) | 100 | Asthma | Not related | Mild | Completed |
| 4 | Positive  (1,280) | 29 | Angioedema | Possibly related | Moderate | Discontinued due to Quincke’s edema and urticaria^†^ |
|  |  |  | Urticaria | Possibly related | Moderate |  |
| **Switch from EU-ref-NTZ to biosim-NTZ at Week 24 (n=1)** | | | | | | |
| 1 | Negative | 169 | Erythema | Related | Mild | Discontinued due to hypersensitivity^†^ |
|  |  |  | Hypersensitivity | Related | Mild |  |
|  |  |  |  |  |  |  |
